# Application of Improved VMD-LSTM Model in Sports Artificial Intelligence

**DOI:** 10.1155/2022/3410153

**Published:** 2022-07-14

**Authors:** Tiancong Zhang, Caihua Fu

**Affiliations:** ^1^School of Physical Education, Sanya University, Sanya 572000, China; ^2^School of Management, Sanya University, Sanya 572000, China

## Abstract

In recent years, with the rapid development of a new generation of artificial intelligence technology, how to deeply apply artificial intelligence technology to physical education and break through the limitations of time-space scenarios and knowledge transfer methods in traditional models has become a key issue in intelligent physical education in the era of artificial intelligence. In order to realize the online monitoring of wearable devices with artificial intelligence in sports and overcome the problem of low recognition accuracy of electrocardiogram, blood oxygen, and respiratory signals in many cases, this paper proposes a combination of variational modal decomposition based on the maximum envelope kurtosis method. Long-short-term neural network (VMD-LSTM) monitoring method for wearable sports equipment. Through experimental analysis and verification, the current signal of the VMD model shows a trend of fluctuating from large to stable and then to large with motion, while the training accuracy of LSTM after the 150th iteration is 94.09%, which shows that the coupling model VMD LSTM can better predict the direction of sports artificial intelligence. In addition, although the training time of the BP neural network is shorter than that of the LSTM model, there is a large gap between the recognition effect and the LSTM, and there are also large differences between different neural network structures. This shows that the VMD-LSTM model has broad application prospects in such models.

## 1. Introduction

Artificial intelligence (Artificial Intelligence, referred to as “AI”) is a discipline that studies how to make machines do things that humans need intelligence to accomplish [1]. Artificial intelligence has experienced the early game, expert system, and other algorithmic models and has developed to the current research direction of machine learning and deep learning [2]. Artificial intelligence is essentially a branch of computer science. With the rapid iterative update of computer technology, it shows stronger intelligence and practicability and is generally accepted by the public. My country's artificial intelligence started late, and there is still a certain gap compared with developed countries such as the United States. Physical education is one of the important contents of the implementation of comprehensive quality education in my country, and it has a basic supporting role in improving the national physique and improving the quality of life [3]. In recent years, the rapid development of a new generation of artificial intelligence technology has had a significant impact on the content, mode, method, and system of traditional physical education [4]. How to deeply apply artificial intelligence technology to physical education and break through the limitations of time-space scenarios and knowledge transfer methods in traditional models has become a key issue in intelligent physical education in the era of artificial intelligence.

In recent years, sports artificial intelligence research, the main subject is computer science, and the algorithm empirical research of basic theory is preferred [5]. It mainly involves the use of neural network-based machine learning-related algorithms, involving sports performance prediction [6], human action recognition and evaluation [7], technical and tactical decision support, sports injury assessment, etc. [8, 9], [10] As the most important research direction in the field of artificial intelligence in recent years, deep learning (Deep Learning) is a kind of intelligent learning algorithm based on deep neural network and representation level, so as to obtain humanlike analytical learning ability, thus promoting the progress of artificial intelligence related technologies [11, 12]. Among them, the human posture recognition technology in deep learning is a technology that can accurately describe the posture and behavior by sensing the structural parameters of human limbs through sensing signals, images, and other data [13]. Detection of human skeleton joints is the core problem of pose recognition [14]. Since 2012, the rapid development of deep learning technology has promoted the continuous improvement of the detection effect of human skeleton joints [15].

Aiming at the lack of error correction design in the traditional Long and Short-term Memory Network (LSTM) structure [16], a sports artificial intelligence based on Variational Modal Decomposition (VMD) was proposed. Intelligent models [17, 18]. Combining the new ideas of “artificial intelligence” with the needs of sports provides a beneficial guarantee for meeting the needs of scientific training. Based on the VMD-LSTM model and the design and use of portable equipment, this paper constructs a sports monitoring service system, which combines a new method of artificial intelligence to analyze the real-time parameters of the sports process. By establishing this service model, the physical activity of athletes can be monitored.

## 2. Principles and Methods

### 2.1. Metamorphic Mode Decomposition

Variational mode decomposition is an adaptive signal processing method proposed in 2014. By iteratively searching for the optimal solution of the variational mode, the modal function and center frequency are continuously updated to obtain several modes with a certain bandwidth function [19]. Variational modal decomposition is an improvement of the EMD algorithm, which has the advantage of better avoiding modal aliasing and end-point effects [20]. Metamorphic Mode Decomposition.

Variational mode decomposition decomposes wearable sports equipment into several eigenmode functions (IMFs) with center frequencies on the premise that each decomposed eigenmode function has a limited bandwidth, by constructing and solving a variational problem to find each eigenmode function and its center frequency [21]. The VMD method decomposes the signal into predefined K eigenmode components IMF, where the IMF is redefined as an AM-FM signal.

(1) ukt=Aktcosϕtk.

The accuracy of the decomposition result of the VMD algorithm depends on the number of modes K and the value of the penalty factor. If the value of K is too large, it will cause excessive signal decomposition, so that a component determined by the center frequency will be scattered in different eigenmode components; while the value of K is too small, it will cause modal aliasing, that is, different centers. The frequency signal components are in the same eigenmode component after being decomposed by VMD, resulting in components that have no practical significance. Both of these cases can lead to inaccurate results of the decomposition. The value of *α* will affect the frequency bandwidth of each modal obtained after the signal is decomposed by the variational modal decomposition algorithm, the degree of modal aliasing and the speed of the algorithm. If the value of the penalty factor *α* is too small, the bandwidth of each frequency is too large, and similar frequencies are mistaken for the large bandwidth part of the same center frequency so that they are decomposed into the same component, and the problem of modal aliasing occurs. When the value is too large, part of the bandwidth is reduced, which may cause the signal to be overresolved and spurious components appear. Therefore, the selection of appropriate parameter values is crucial to the accuracy of the PPG signal decomposition results [22].

### 2.2. LSTM Model

Long Short-Term Memory (LSTM) is a temporal recurrent neural network [2]. The outstanding contribution of this network lies in the use of the self-loop design, and the weight of the self-loop can also be updated in each loop iteration, thus cleverly avoiding the gradient dispersion problem that occurs when the recurrent neural network model updates the weights [23].

**Figure 1:**
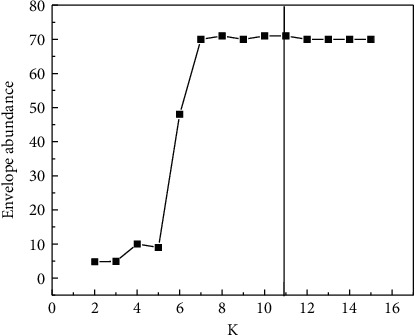
Variation Trend of maximum envelope kurtosis.

Each hidden node of the LSTM network model contains a storage unit, and the entrance of the storage unit is controlled by some logic units. These control units are generally called “gates.” At the entrance of each hidden unit, there are three gates, namely, input gate, output gate, and forget gate [24]. The input gate is used to selectively save the input data, the output gate is used to determine when to output the value, and the forget gate is used to determine when the value should be memorized or forgotten [25]. Hidden nodes are shown in Figure 1. In the whole model, the parameters of the input gate, output gate, and forget gate in each hidden node are learned during the training process [26]. The calculation formulas for the three gates are as follows:

(2) $$\begin{array}{c} \;i_{t}=\sigma \left(W_{\mathrm{xi}}x_{t}+W_{\mathrm{hi}}h_{t-1}+b_{i}\right),\\ o_{t}=\sigma \left(W_{\mathrm{xo}}x_{t}+W_{\mathrm{ho}}h_{t-1}+b_{o}\right),\\ f_{t}=\sigma \left(W_{\mathrm{xf}}x_{t}+W_{\mathrm{hf}}h_{t-1}+b_{f}\right),\\ \;c_{t}=f_{t}c_{t-1}+i_{t}tanh\left(W_{\mathrm{xc}}x_{t}+W_{\mathrm{hc}}h_{t-1}+b_{c}\right),\\ h_{t}=o_{t}tanh\left(c_{t}\right). \end{array}$$

## 3. Experimental Procedure and Result Analysis

### 3.1. Feature Extraction Using VMD Method

The maximum envelope kurtosis method is used to determine the optimal decomposition level K of the VMD, randomly extract several groups of current signals from the first group, and use the maximum envelope kurtosis method for analysis. By observing the variation trend of the local maximum envelope kurtosis in Figure 1, it can be found that the envelope kurtosis is the largest when *K* = 11.

So take *K* = 11 VMD for current signal processing. It can be seen from the decomposed current signal that IMF1, IMF2, and IMF3 are low-frequency signals, the frequency components are relatively single, and the main frequency components of the current signal are concentrated in the low-frequency current signal. Therefore, the features of the first three IMFs can be directly extracted to represent the movement and detect changes in parameters. There are interference signals such as noise in the high-frequency signal of the current signal, and after calculation, the signal-to-noise ratio in the high-frequency signal is less than 0, which is dominated by noise, and the soft threshold denoising method needs to be used for denoising. The original signal can be regarded as the sum of the decomposed IMFs, so the high-frequency signal can be simplified as follows:

(3) $$\begin{array}{c} {\text{IMF}}_{h}=\sum_{i=4}^{11}{\text{IMF}}_{i}\;i=4,5,\ldots ,15. \end{array}$$

It can be seen from the calculation that the amplitude of the curve of the denoised signal is smaller than that of the high-frequency signal, which means that some noise is removed and the characteristics of the tool wear signal are preserved. The signal-to-noise ratio of the high-frequency signal before denoising is 6.581 dB, and the signal-to-noise ratio after denoising is improved to 2.024 dB.

Since it is difficult to find the relationship between the signal and sports parameters by directly observing the signal, the original signal is processed to extract features that are related to the body motion parameters. This paper extracts 7-dimensional time domain features, including mean, root mean square, peak value, standard deviation, variance, and kurtosis factor, margin factor; 2-dimensional frequency domain feature, including barycentric frequency and frequency energy value, extracting the mean, root mean square, and peak value of 3 low-frequency IMFs, and one high-frequency IMF, a total of 21 dimension features. Since the classification of sports parameters under multiple working conditions in this paper needs to consider the influence of parameters such as heart rate on the current signal, the three sports parameters are also classified as features, with a total of 24-dimensional features. The model training speed will be slowed down due to too many feature dimensions, and there may be features that are not related to motion parameters in these features. In this paper, the SelectKBest class in the feature-selection of the Python library is combined with the mutual information method to filter the features, and the 6-dimensional features plus 3 cutting parameters are selected from the 21-dimensional feature parameters as the model input.

### 3.2. Validation of Model Recognition Effect

**Figure 2:**
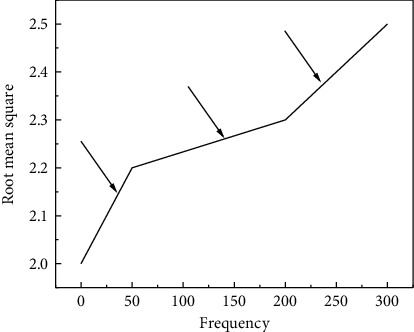
Trend chart.

A total of 921 groups of experimental data were collected in this experiment, and the number of experimental data in each group. Taking the first group as an example, the current signal is divided into three stages, the initial stage of exercise, the middle stage of exercise, and the later stage of exercise. The variation trend of the current signal eigenvalues with motion is shown in Figure 2.

It can be observed from Figure 2 that in the initial stage, the current signal fluctuates greatly, because the body movement state is good, and the body function parameters have not yet adapted to the body. In the normal stage, the current change is relatively gentle, and in the severe stage, it rises sharply, which is caused by the sudden strengthening of the exercise state after the exercise adaptation.

### 3.3. Normalization

Since there are few initial and late samples in each group of experiments, normal samples occupy the vast majority, and unbalanced samples will affect the final classification effect. Therefore, the SMOTE algorithm is used to oversample the interval with fewer samples. This method is used in minority samples. New sample points are generated on the Euclidean distance [27]. After dividing the 4 groups of experimental data into 3 intervals, the training set and the test set are divided according to the ratio of 3:1. For the multiclassification model, the output label corresponding to each state needs to be coded. In this paper, the One-Hot coding method is used. The data set composed of features after optimization needs to be normalized before classification, and the normalization formula is

(4) $$\begin{array}{c} x=\frac{x-x_{\text{min}}}{x_{\text{max}}-x_{\text{min}}}. \end{array}$$

As a variant of Recurrent Neural Network (RNN), LSTM can better avoid the problem of gradient disappearance and can effectively deal with the long-term dependency problem [1]. Due to the small number of samples collected, this paper designs a model with only a single LSTM layer, the number of LSTM neural units is set to 30, the batch size is set to 32, the cross entropy is selected as the loss function, and the Adam optimization algorithm is used to optimize the model parameters [28], and the structure of the LSTM model is shown in Table 1.

**Figure 3:**
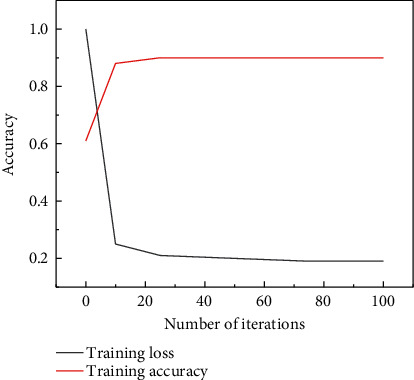
Model accuracy training and loss curve.

Figure 3 shows the accuracy and loss curve of the LSTM model training process. It can be observed from the figure that the training accuracy value and loss tend to converge after the 150th iteration, and the training accuracy during the training process reaches 94.09%. The classification accuracy rate on the test set reached 92.44%. The loss rate is another way of expressing the accuracy. The loss rate in Figure 3 is stable at about 10% after multiple iterations, which indicates that the model selected in this study has good accuracy and strong applicability. It can be concluded that the model has high recognition accuracy and strong generalization ability, which can meet the recognition requirements of actual motion state parameters. The selected features can better characterize the change of motion state, which is similar to the research results of some scholars [29].

In order to verify the superiority of the model in this paper for the recognition of motion states under multiple working conditions, this paper uses empirical mode decomposition (EMD) and ensemble empirical mode decomposition to construct different feature vectors for the same sample set [28] and uses the LSTM model for Classification [30], the final result is far from the method in this paper, and the accuracy is not high. And, this paper compares the LSTM model with the traditional machine learning method Support Vector Machine (SVM) and BP neural network, in which the kernel function parameter *σ* and penalty factor C of SVM use particle swarm optimization algorithm to find the best parameters, respectively, are 11.580 and 12.926. The BP neural network adopts the structure of 30 × 8 × 3, and the number of iterations is 500 times. In order to avoid experimental chance, each model was tested 5 times to take the average of the accuracy of the test set.  Table 2 shows the test results of the model.

It can be seen from the table that the training time of SVM is longer than that of LSTM, and the accuracy of the test set is lower than that of LSTM. The data samples in this paper are changed from 921 to 1851 groups after sample synthesis. Efficiency is lower. Although the training time of the BP neural network is shorter than that of the LSTM model, there is a big gap between the recognition effect and LSTM, and there are also big differences between different neural network structures, which is similar to the research results of some scholars [31].

## 4. Model Predictions

In the research on the physical health of different groups of people, intelligent wearable devices based on machine learning and deep learning algorithms can identify, monitor, and analyze the muscular endurance and physical activity status of people with different physical fitness, including children, adolescents, and adults. Proposing exercise methods suitable for individuals with different physical conditions can promote the formation of healthy living habits.

In the monitoring test, there are many factors that affect the effect of sports wearable devices, including respiratory rate, heart rate, and real-time measurement of hemoglobin. The measurement of respiratory rate is affected by detection time and exercise intensity. Therefore, to obtain accurate respiratory frequency measurement, it is necessary to remove the influence of clutter on the respiratory measurement results. The least mean square can solve the respiratory frequency well, so the system decides to use the least mean square respiratory signal filtering algorithm as the main technology of this module. If all the influencing factors are considered, the experiment becomes very complicated and requires a huge cost. Therefore, the simplified variables in this paper are the fluctuation of electrocardiogram, respiratory signal, and monitoring of blood oxygen concentration.

**Figure 4:**
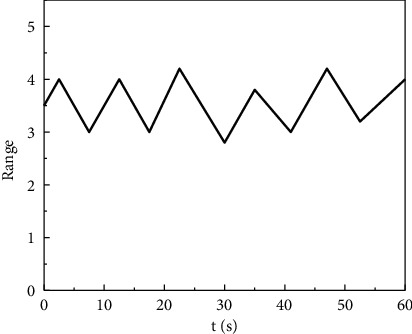
Respiratory signal after filtering respiratory signal using least mean square.

The electrocardiogram is an electrical activity process that reflects the excitement of the heart. It has important medical reference significance and reflects the activity process of the human body's electrocardiogram. It has considerable medical and sports measurement importance. In the ECG monitoring part, the system uses textile electrodes as the main material, because when the material contacts the skin, an equivalent capacitance C1 will be generated, which can achieve signal impedance matching and improve the sensitivity and accuracy of signal detection. The single-lead ECG measurement module made of textile electrodes is placed at two points on the surface of the human body. By measuring the potential difference between the two points, the signal can be transmitted to the analog ECG signal conversion interface through the wire, as shown in Figure 4.

The respiratory signal spectrum after filtering processing has regular breathing fluctuation as a whole, and the fluctuation is stable. The maximum amplitude of the fluctuation is 1.5 V, which occurs between 21 and 29 seconds. Within seconds, it can be seen that the monitored exercise participant's breathing is stable, the exercise training intensity is reasonable, and there is no need to adjust the amount of exercise. The least mean square can solve the respiratory frequency well, so the system decides to use the least mean square respiratory signal filtering algorithm as the main technology of this module. The least-mean-square respiratory signal filtering algorithm consists of five operating modules, firstly measuring *n* times of inhalation signal *x*, then measuring *n* times of exhalation signal *e*, and then adding the two different signals to calculate the sum signal frequency value, then the three-axis acceleration and the breathing signal are input into the digital filter together, and the breathing signal processed by the digital filter is then output for correlation detection. In square algorithm operation, the operation result will be input into the digital filter again, so as to reciprocate until the error is wirelessly reduced and the error is corrected continuously, so as to obtain the accurate respiratory data measurement signal. Capture, the process of data capture is the real-time measurement of human body functions such as heart rate and hemoglobin by wearable devices, so the monitor of the wearable device becomes the key device for this step.

## 5. Conclusion

Based on the perspective of the era of intelligence, on the basis of traditional sports and with the help of deep learning technology in the field of artificial intelligence, this research proposes an intelligent evaluation algorithm for sports wearable devices. Sports smart wearable system. In order to realize the online monitoring of wearable devices with artificial intelligence in sports and overcome the problem of low recognition accuracy of electrocardiogram, blood oxygen, and respiratory signals in many cases, this paper proposes a combination of variational modal decomposition based on the maximum envelope kurtosis method. Long-short-term neural network (VMD-LSTM) monitoring method for wearable sports equipment. Through experimental analysis and verification, the following conclusions are drawn: (1) in the early stage of the exercise, the current signal fluctuates greatly, and the current changes relatively gently in the normal stage, and then rises sharply in the severe stage; (2) after the 150th iteration, the training accuracy value, and the loss tends to converge, in which the training accuracy reached 94.09% during the training process, and the classification accuracy of the model on the test set reached 92.44%; (3) the training time of SVM is longer than that of LSTM, and the testing time is longer than that of LSTM. The accuracy rate of the set is lower than that of LSTM, and the efficiency of using SVM is relatively low; (4) although the training time of the BP neural network is shorter than that of the LSTM model, the recognition effect has a large gap with LSTM, and there are also differences between different neural network structures.[32], [33].

## Figures and Tables

**Table 1:** Inspection and test results of external wall damage.

LSTM model structure	Neurons of each layer quantity	Incentive at all levels function	Input of each layer dimension<
Input layer	Feature dimension	—	(None, 1)

LSTM layer	30	Sigmoid	(None, 30)

Full connection layer	8	ReLu	(None, 8)

Output layer	3	Softmax	(None, 3)

**Table 2:** Model test results.

Classification model	Test set average classification accuracy (%)	Optimal classification of test set accuracy (%)	Train time (S)
LSTM	93.78	94.02	83.35
PSO-SVM	90.43	91.67	89.49
BP neural network	88.59	89.21	39.86

## Data Availability

The data used to support the study are included in the paper.
